# Science communication challenges about antimicrobial resistance in animal agriculture: insights from stakeholders

**DOI:** 10.1093/jacamr/dlac032

**Published:** 2022-03-29

**Authors:** Andy J. King, Dara M. Wald, Denise D. Coberley, Michael F. Dahlstrom, Paul J. Plummer

**Affiliations:** 1 Greenlee School of Journalism and Communication, Iowa State University, Ames, IA, USA; 2 National Institute of Antimicrobial Resistance Research & Education, Ames, IA, USA; 3 Department of Agricultural Leadership, Education and Communications, Texas A&M University, College Station, TX, USA; 4 Applied Linguistics Program, Department of English, Iowa State University, Ames, IA, USA; 5 College of Veterinary Medicine, Iowa State University, Ames, IA, USA

## Abstract

**Background:**

Communicating about antimicrobial resistance (AMR) and antimicrobial stewardship (AMS) requires technical knowledge, consideration of audience values and appropriate identification of communication strategies for multiple audiences. Within the context of animal agriculture, communicating about AMR represents an important and complex endeavour for veterinarians, governmental agencies, producers and the industry to convey policy and practice information regarding the use of antimicrobials in food animals.

**Objectives:**

To assess the science communication challenges related to AMR by identifying the motivations, goals and struggles of animal agriculture stakeholders when communicating about AMR and AMS.

**Methods:**

Participants attending a meeting on AMR communication in animal agriculture (*N = *80) completed a workshop on science communication, including small group meetings with oral/written comments collected. Participants included veterinarians, government agency representatives, industry stakeholders and producers.

**Results:**

Results indicated participants believed providing more accurate information would resolve misunderstanding and concern about AMR to other stakeholders, counter to recommendations of science communicators. Other participants noted beliefs about the utility of stories in trying to explain how AMS is normative and consistent with the values of all parties interested in animal agriculture. Participants noted the importance of public engagement, even if the participants’ perceived target audiences did not include the public.

**Conclusions:**

Communicating about AMR and AMS in animal agriculture contexts provide unique challenges. Few evidence-based recommendations are available for science communicators in these contexts and more research is needed to improve the quality of communication about AMR and AMS in animal agriculture.

## Introduction

Domestic and international public health agencies have identified antimicrobial resistance (AMR) as an urgent public health threat.^1,2^ AMR results in over $2 billion of human healthcare costs annually in the USA.^3^ Research on AMR infections, and how to talk about AMR as a public health threat,^4,5^ focuses on ‘general public’ understanding of AMR effects on human health with little attention to perspectives of animal agriculture stakeholders.^6^ Given that considerable antibiotic use occurs in animal agriculture,^7^ there is a clear need to better understand how animal agriculture stakeholders communicate about antimicrobial stewardship (AMS)—the use of antimicrobials based on ‘optimizing therapeutic efficacy while minimizing risk of antimicrobial resistance’.^8^

One challenge of considering communication efforts among animal agriculture stakeholders is the diversity of invested stakeholder groups to consider. Farmers and producers, those who manage and run operations where animals may be treated with antibiotics, may perceive their stakeholders to be retailers and consumers who buy their product, veterinarians who treat their animals and government officials who might draft regulations producers must follow. Each of these stakeholder groups has unique obligations and limitations related to AMR. For example, veterinarians must adhere to regulations from government agencies, discuss treatment plans for clients and serve as a public-facing group given the visibility of their profession to mainstream audiences. Similar complexities exist for other animal agriculture stakeholders whose diverse obligations, audiences and communication goals require varied, and effective, science communication strategies. At present, few studies examine how the many stakeholder groups in animal agriculture manage and address communication challenges relevant to AMR.^6,9^

The current study reports the outcomes of an interactive, in-person workshop focused on improving science communication about AMR among animal agriculture stakeholders. The objective of the workshop evaluation reported here was to increase understanding about the communicative challenges facing animal agriculture stakeholders and provide a foundation for improving AMR communication efforts in the future.

## Methods

Participants in this study were science communication workshop attendees at a national antibiotic symposium hosted by the National Institute of Animal Agriculture. The workshop included participants representing five stakeholder groups (see Table 1). The authors facilitated an activity where participants (*N = *80) responded to prompts about (i) why communication about AMR is important to their job; (ii) the audience with which they wanted to better communicate about AMR; (iii) a challenging AMR communication situation faced in the past; (iv) the cause of said communication challenge; and (v) how to improve communication efforts in the future.

**Table 1.** Participant information

Category	*n* (%)
Gender	
Male	47 (59)
Female	33 (41)
Years in industry, mean	16.37
Stakeholder group	
Veterinarian	31 (39)
Industry	25 (31)
Producer/farmer	10 (13)
Government	22 (28)
Academic/research	20 (25)

*N = *80. Years in industry ranged from under 1 year to ‘life’; the average was calculated from responses provided numerically (*n = *77, median = 15 years). Stakeholder groups do not add up to 100% as participants were allowed to select multiple categories. Participants in the ‘Government’ category included researchers and those involved in policymaking processes.

Participants first wrote their own answers to each prompt and then engaged in observed group conversations about written responses and other experiences in communicating about AMR. We summarize the themes and ideas discussed by the stakeholder groups in the current paper based on thematic coding^10^ of the written responses and group discussion notes. See Table 1 for participant information. The stakeholder group with the smallest representation in the study was producers and farmers.

## Results

Full results appear in Table 2. We provide observations about convergent themes below.

**Table 2.** Response themes for the study prompts stratified by stakeholder group

Stakeholder group	Why AMR communication is important to your job	With which audiences do you want to improve AMR communication?	Challenging communication situations around AMR	Perceived cause of AMR communication challenges	Beliefs about improving future AMR and AMS communication
Veterinarian	Clarify public and client misperceptions about the complexities of antimicrobial use Vets are gatekeepers of antibiotics and viewed as thought leaders	Consumers/public Producers/farmers Students Policy influencers (i.e. advocacy groups and politicians)	Uncertain knowledge of audience (e.g. maybe nothing or skewed by experience) Fighting misperceptions about current policies and antibiotic use	Ineffective delivery of complex and nuanced information Emotional reactions and impatience (by communicator or receiver)	Stories, examples, and analogies Accurate non-technical information Active listening first, then information presentation
Industry	Consumer confidence (e.g. share knowledge about food safety and supply chain) Influence policy to avoid additional regulation on use	Consumers/public (includes meat buyers) Producers Regulatory agencies	Lack of knowledge about production, food supply chain, and labelling Communicating with groups believed to have existing agendas or that focus more on emotions than facts	Lack of available consumer-friendly language to talk about AMR complexities Myths and media misrepresentation of industry and practices (includes social media)	Positive stories about industry practices (e.g. focused on safe food supply) Disseminate accurate information and misinformation corrections Find common ground with public
Producer/farmer	Defend ability to protect animal welfare (e.g. to government officials) Educate consumers for marketability	Consumers/public Those involved with policy and regulation (media, politicians, regulators, etc.)	Consumer misunderstanding about antibiotic use and labelling Unfamiliarity with animal agriculture (e.g. how and why antibiotics are used)	Hostility about animal agriculture practices from some consumers and advocacy groups Lack of consumer knowledge	Improve consumer education about animal agriculture Use ‘more facts’ and reputable sources Appeal to animal welfare implications through storytelling
Government	Public perceptions can drive legislation and regulation (helpful and unhelpful) Important for biosecurity, as well as animal and human health Educating producers	Consumers/public (‘non-scientists’ and voters) Producers/industry groups Legislators and other government agencies	Technical details (residue versus resistance; antibiotics needed for animal welfare) Focusing on takeaway messages and not process for some audiences Difficult to quantify return on investment on AMR prevention efforts	Misunderstanding of the science of AMR Inconsistent messaging across stakeholder groups Lack of audience trust Stakeholders contest claims of expertise	More explanation from trusted sources Appeal to audience values and emotions Focus on opportunities to interact with audiences and find common ground
Academic/research	Obligation to promote stewardship to stakeholders Need to reach multiple sectors with AMR-relevant information	Consumers/public Students/youths Producers/farmers Extension educators Regulatory agencies Politicians	Creating messages that resonate Having interactions with people across sectors (including human health) Discussing regulations with producers	Different knowledge bases for a complex topic Focusing on precision instead of clarity Lack of rapport and different values	Be precise and accurate, and focus on education Ask questions (and listen) to determine what to talk about Use storytelling

### Why is communication about AMR important?

The stakeholder groups differed most frequently in responding to this question. Responses ranged from a need to enhance consumer confidence, influence policy, protect animal welfare, expand public understanding and promote stewardship. One convergent area of consideration was the need to have antibiotics as a treatment option for disease care and animal welfare. There was also concern about misunderstanding and misinformation about antibiotic use in animal agriculture (and animal health more broadly) by the public across multiple (but not all) stakeholder groups.

### Which audience should be the focus of improved AMR communication?

All stakeholder groups indicated the need to improve consumer/public understanding of AMR and AMS at varying levels of priority. Policymakers (e.g. politicians, agency personnel) were the only other audience segment of interest mentioned by most groups.

### What are challenges to communication about AMR?

Most participants, across groups, indicated a lack of knowledge (of some audience) hindering communication attempts. Unfamiliarity with animal agriculture practices and policies was another frequently cited source of communication challenges.

### What was the perceived cause of AMR communication challenges?

Participants noted inconsistent messaging, the complexities of AMR policies and concerns, and lack of consumer knowledge as drivers of communication challenges. Other reasons for challenges included beliefs about media messages influencing myths and misperceptions and that animal agriculture messaging often focuses more on precision/accuracy than clarity.

### What could improve future AMR/AMS communication?

Many participants believed using stories and analogies focusing on shared values (e.g. animal welfare) and emotions might be a useful approach. Other participants advocated for a deficit model approach, relying on the provision of information to the target audience to correct a perceived knowledge deficit. While this strategy remains a popular and oft-cited approach for public outreach, it has been found to be consistently *ineffective* in science communication.

## Discussion

The current study suggests that agricultural stakeholders are aware of the considerable challenges in communicating about AMR to audiences of interest. Many responses for improving communication aligned with strategies that science communicators advocate (e.g. stories, analogies, using trusted sources, appealing to values/emotions of audiences of interest). However, there were still participants who favoured simply filling the perceived gap in knowledge related to AMR and AMS, which science communication research has shown repeatedly to be unproductive. Findings from this study suggest a need for more engagement between animal agriculture stakeholder groups and social scientists who could provide recommendations/training about communication and public engagement theory and practice that move beyond the deficit model. Moreover, the breadth of opinions and issues addressed by these stakeholders highlights the need for more nuanced approaches to communication/outreach with a diversity of stakeholder groups with animal agriculture interests. Without a larger evidence base, AMR communication will continue to be a challenge, hindering progress toward widespread adoption of AMS behaviours. Evidence-based recommendations will improve communication and facilitate behaviour change that contributes to AMS efforts across animal health contexts.
